# Genetically engineered CD80–pMHC-harboring extracellular vesicles for antigen-specific CD4^+^ T-cell engagement

**DOI:** 10.3389/fbioe.2023.1341685

**Published:** 2024-01-17

**Authors:** Irina A. Ishina, Inna N. Kurbatskaia, Azad E. Mamedov, Elena I. Shramova, Sergey M. Deyev, Kamila S. Nurbaeva, Yury P. Rubtsov, Alexey A. Belogurov, Alexander G. Gabibov, Maria Y. Zakharova

**Affiliations:** ^1^ Shemyakin-Ovchinnikov Institute of Bioorganic Chemistry of the Russian Academy of Sciences, Moscow, Russia; ^2^ Biomarker Research Laboratory, Institute of Fundamental Medicine and Biology, Kazan Federal University, Kazan, Russia; ^3^ Sechenov First Moscow State Medical University, Sechenov University, Moscow, Russia; ^4^ V. A. Nasonova Research Institute of Rheumatology, Moscow, Russia; ^5^ N. N. Blokhin National Medical Research Center of Oncology of the Ministry of Health of the Russian Federation (NN Blokhin NMRCO), Moscow, Russia; ^6^ Department of Biological Chemistry, Evdokimov Moscow State University of Medicine and Dentistry, Moscow, Russia; ^7^ Department of Life Sciences, Higher School of Economics, Moscow, Russia; ^8^ Department of Chemistry, Lomonosov Moscow State University, Moscow, Russia; ^9^ Pirogov Russian National Research Medical University, Moscow, Russia

**Keywords:** CD4^+^ T cells, extracellular vesicles, major histocompatibility complex, costimulatory molecules, antigen-specific expansion

## Abstract

The identification of low-frequency antigen-specific CD4^+^ T cells is crucial for effective immunomonitoring across various diseases. However, this task still encounters experimental challenges necessitating the implementation of enrichment procedures. While existing antigen-specific expansion technologies predominantly concentrate on the enrichment of CD8^+^ T cells, advancements in methods targeting CD4^+^ T cells have been limited. In this study, we report a technique that harnesses antigen-presenting extracellular vesicles (EVs) for stimulation and expansion of antigen-specific CD4^+^ T cells. EVs are derived from a genetically modified HeLa cell line designed to emulate professional antigen-presenting cells (APCs) by expressing key costimulatory molecules CD80 and specific peptide–MHC-II complexes (pMHCs). Our results demonstrate the beneficial potent stimulatory capacity of EVs in activating both immortalized and isolated human CD4^+^ T cells from peripheral blood mononuclear cells (PBMCs). Our technique successfully expands low-frequency influenza-specific CD4^+^ T cells from healthy individuals. In summary, the elaborated methodology represents a streamlined and efficient approach for the detection and expansion of antigen-specific CD4^+^ T cells, presenting a valuable alternative to existing antigen-specific T-cell expansion protocols.

## Introduction

Tracking antigen-specific T cells is indispensable for understanding the cellular immune response and evaluating the efficacy of antigen-specific immunotherapies across a broad spectrum of conditions, including viral infections, cancer, and autoimmune diseases (Serra and Santamaria, 2019; Hont et al., 2022). CD4^+^ T cells play a pivotal role in orchestrating adaptive immune responses through activating innate immune cells, B cells, cytotoxic CD8^+^ T cells, and non-immune cells and exerting regulatory functions (Luckheeram et al., 2012; Cui et al., 2021; Marks and Rao, 2022). These multifaceted functions depend on the recognition of specific antigenic peptides presented in complex with MHC-II by the T-cell receptor (TCR). However, antigen-specific CD4^+^ T cells, particularly those specific to neoantigen or autoantigen peptides, are rarely frequent within peripheral blood mononuclear cells (PBMCs), posing a technical challenge for the direct examination of their phenotype (Bacher et al., 2013).

Various technologies have emerged for the detection of antigen-specific CD4^+^ T cells, among which the multimerization of fluorescently labeled pMHCs has become a prominent method (Altman et al., 1996; Crawford et al., 1998). Tetramer technology, leveraging cytometry-based methods, has enabled the identification of antigen-specific CD4^+^ T-cell populations implicated in diverse immune-mediated diseases (Christophersen et al., 2019; Patel et al., 2022; Sharma et al., 2023; Vyasamneni et al., 2023). While high-throughput tetramer peptide re-loading has been achieved through linker cleavage (Willis et al., 2021), its applicability is limited due to the instability of certain MHC-II alleles during this modification. Notably, certain pMHC tetramers exhibit limitations in staining rare or low-affinity CD4^+^ T cells. To address this, affinity maturation to CD4 molecules has been developed, albeit with testing limited to a specific subset of MHC-II alleles (Sugata et al., 2021). Alternatively, to enhance avidity for specific TCRs, MHC-II dextramers and dodecamers have been developed (Dolton et al., 2014; Huang et al., 2016). Given the challenges associated with tetramer production, various approaches are continually evolving. Among these approaches, the use of pMHC microarrays stands out for detecting antigen-specific CD4^+^ T cells (Soen et al., 2003; Ge et al., 2010). However, the application of array techniques demands a substantial quantity of target cells, presenting challenges in managing flow conditions and achieving the appropriate orientation of pMHC on the array surface. These challenges impose constraints on both sensitivity and specificity (Bentzen and Hadrup, 2017). Antigen-specific CD4^+^ T cells were isolated using an alternating-current electrohydrodynamic-based microfluidic platform combined with surface-enhanced Raman scattering (Dey et al., 2019). Additionally, proximity labeling approaches have proven successful in detecting CD4^+^ T cells (Liu et al., 2020; Liu et al., 2022). However, antigen-specific pre-enrichment may prove beneficial for achieving more sensitive detection and comprehensive characterization of antigen-specific CD4^+^ T cells.

Professional antigen-presenting cells (APCs), specifically conventional dendritic cells (DCs), possess unparalleled potency in activating T cells due to their phagocytic capabilities and the expression of diverse costimulatory molecules. Owing to their functionality, monocyte-derived DCs (MoDCs) are the most widely used cell population for antigen-presentation purposes and antigen-specific expansion (Patente et al., 2019; Cauwels and Tavernier, 2020; Gu et al., 2020). However, the generation of MoDCs entails prolonged differentiation and maturation procedures, often resulting in an inadequate yield of functional DCs (Steenblock et al., 2009). The potency of T-cell stimulation relies on the high density of pMHC presentation, closely mimicking the role of the secondary lymphoid organs as the primary sites for naïve T-cell antigenic engagement (González et al., 2005). To circumvent the drawbacks associated with the use of cells and create a high-density antigenic environment, various cell-free systems employing nano/micro-particles have been developed (Singha et al., 2017; Rhodes et al., 2021; Isser et al., 2022).

EVs, encompassing ectosomes or endosomes, based on their size, are membrane-enclosed vesicles secreted by all cell types. EVs play a crucial role in intercellular communication, boasting targeting specificity and physical stability (Yáñez-Mó et al., 2015). The compartmentalization of immunostimulant molecules in EVs becomes an efficient tool in novel immunotherapeutic approaches (Ukrainskaya et al., 2021; 2023). Analogous to other cells, APCs secrete EVs carrying replicas of their cell surface, including pMHC and costimulatory molecules (Lindenbergh et al., 2020). The generation of EVs necessitates prolonged cultures and isolation procedures. Several approaches, including sonication, yield EV-like particles of exosomal size. Although the size of EVs aligns with engineered particles, the density of pMHCs may be insufficient when employing EV-like nanoparticles (Vincent-Schneider et al., 2002).

Herein, we report the generation and application of EVs derived from a genetically modified HeLa cell line expressing CD80 and pMHC of interest by routine cytochalasin B treatment and further benchtop centrifugations. Our data unequivocally show that antigen-presenting EVs exhibit a size similar to microvesicles and carry a sufficient amount of antigen to activate and subsequently induce proliferation of antigen-specific CD4^+^ T cells.

## Materials and methods

### Cells and culturing conditions

HEK293T and HeLa were provided by Bioresource collection—Collection of SPF-Laboratory Rodents for Fundamental, Biomedical and Pharmacological Studies supported by the Ministry of Science and Higher Education of the Russian Federation (Contract No. 075-15-2021-1067). Cell lines were cultured in media (DMEM or RPMI 1640) supplemented with 10% FBS, 100 U/mL penicillin, 100 μg/mL streptomycin, 0.25 μg/mL amphotericin B, and 2 mM GlutaMAX (Gibco). The HEK293T and HeLa cell lines were cultured in DMEM. The Jurkat 76 TPR cell line was cultured in RPMI 1640. Human PBMCs were isolated from the blood of healthy donors by gradient density centrifugation on a Ficoll-Paque (GE Healthcare) according to the standard protocol. Healthy donors provided informed consent. The cell lines were tested for the presence of *mycoplasma* contamination (Evrogen).

### Constructs

Synthetic genes were amplified and cloned into a suitable vector (Supplementary Table S1). TCR amplicons of alpha and beta chains were produced by overlapping PCR. CD80 and TCR amplicons were restricted with XbaI/BamhI and cloned into the pLV2 lentiviral vector (Clontech) under the control of the EF1a promoter (Supplementary Figure S1). Each sequence of the peptide linked to MHC-II was produced by PCR, restricted with NheI/BamHI, and ligated into the pLV2 vector carrying the MHC-II leader sequence. The CD4 amplicon was restricted with NheI/BamHI and ligated into the lentiviral pLX301 vector under the control of the CMV promoter.

### Antibodies

The following fluorophore-conjugated antibodies were used: anti-human CD80-PE (clone W17149D, BioLegend), anti-human HLA-DR-APC (clone L243, BioLegend), anti-human CD4-APC-Alexa Fluor 750 (clone S3.5, Thermo Fisher Scientific), anti-human CD3-APC or CD3-FITC (clone OKT3, BioLegend), anti-human TCR α/β-APC (clone IP26, BioLegend), anti-human CD69-APC (clone FN50, BioLegend), anti-human IFNγ-PE (clone 4S.B3, BioLegend), and anti-human CD11c-FITC (clone 3.9, BioLegend).

### Transduction of Jurkat 76 TPR and HeLa cell lines

Lentiviral particles were produced by PEI co-transfection of HEK293T cells with plasmids encoding genes of interest and packaging plasmids. The medium was changed after 6 h post-transfection to OptiMEM, supplemented with GlutaMAX, sodium pyruvate, and 5% FBS (Gibco). The supernatant was collected after 48 h post-transfection and filtered. The medium containing lentiviral particles was added to the cell line in a 6-well plate and centrifuged at 1200 *g* at 30°C for 90 min with the addition of polybrene (Merck) at 10 μg/mL. The following day, the culture medium was changed to the usual medium. After 3 days post-transduction, CD4^+^ Jurkat 76 TPR cells were selected with 1 μg/mL puromycin (InvivoGen) for 7 days.

### Generation of EVs

EVs were obtained following a published protocol with some modifications (Ukrainskaya et al., 2021). The HeLa cell line was detached from the flask using 5 mM PBS-EDTA for 10 min at 37°C and 5% CO_2_. Cells were centrifuged and re-suspended in DMEM, 10% FBS, and 1% pluronic F-127 (Merck) supplemented with cytochalasin B (Merck) at a concentration of 10 μg/mL. Cells were incubated for 30 min at 37°C with 5% CO_2_ at a density of 1 × 10^6^ cells/mL in a T-25 flask (Corning). Then, cells were shaken intensively for 30 s for the detachment of EVs and centrifuged at 100 *g* for 10 min at 4°C. The supernatant was collected and centrifuged at 300 *g* for 10 min. The resulting supernatant was centrifuged at 3500 *g* for 30 min for EV precipitation. EVs were counted using the NovoCyte flow cytometer (ACEA Biosciences) and frozen at −80°C.

### Immunostaining of cells and EVs

Cells were washed with PBS and re-suspended in PBS with fluorescent antibodies. HeLa cells were stained with anti-human CD80 and anti-human HLA-DR. Jurkat 76 TPR cells were stained with anti-human CD4, anti-human CD3-APC, and anti-human TCR. The staining was performed for 30 min at 4°C. Then, cells were washed with PBS and analyzed using the NovoCyte flow cytometer (ACEA Biosciences). Centrifuging steps were performed at 300 g at 4°C.

EVs were washed with DMEM, 10% FBS, and 1% pluronic F-127 and re-suspended in the same medium with fluorescent antibodies anti-human CD80 and anti-human HLA-DR. The staining was performed for 30 min at 4°C. Then, EVs were washed with the same medium and analyzed using the NovoCyte flow cytometer (ACEA Biosciences). Centrifuging steps were performed at 5000 g at 4°C.

### Confocal microscopy

HeLa cells or HeLa-derived EVs were applied to a poly-L-lysine-covered 96-well imaging glass plate (Eppendorf) and centrifuged at 100 g for 10 min at room temperature. The attached cells and EVs were fixed in 4% paraformaldehyde in PBS for 1 h at room temperature and stained with Hoechst 33342 (Invitrogen), anti-human CD80-PE, and anti-human HLA-DR-APC antibodies. Confocal images were captured using a laser scanning microscope LSM 980 (ZEISS) with a ×63 oil Plan-Apochromat objective (numerical aperture 1.4). Hoechst 33342 was exited at 405 nm, and the emission was detected in the range of 410–605 nm; PE fluorescence was exited at 543 nm, and the emission was registered in the range of 543–623 nm; APC fluorescence was excited at 639 nm, and the emission was detected in the range of 569–694 nm.

### Jurkat 76 TPR activation analysis

Jurkat 76 TPR cells (100,000 cells per well) were incubated with EVs or MoDCs at different T cell:EV or MoDC ratios (2:1, 1:1, 1:2, and 1:5) for 16 h in a 96-well flat bottom plate (Corning). Positive control cells were incubated with 50 ng/mL PMA (Merck) and 1 μg/mL ionomycin (Merck). The volume of each well was 200 μL. Following incubation, cells were washed and analyzed for GFP expression using the NovoCyte flow cytometer (ACEA Biosciences). Alternatively, Jurkat 76 TPR cells were analyzed for the CD69 activation marker. Jurkat 76 TPR cells were incubated with EVs under the same conditions, stained with anti-human CD69, washed, and analyzed using the NovoCyte flow cytometer (ACEA Biosciences).

### T-cell *in vitro* stimulation or expansion and intracellular IFNγ staining

CD4^+^ T-cell isolation from PBMCs was performed following the manufacturer’s protocol (STEMCELL Technologies). CD4^+^ T cells were incubated with EVs at a 1:1 ratio in AIM-V medium (Gibco) supplemented with AlbuMAX (Gibco) in a 96-well round bottom plate. After 3 h of incubation, brefeldin A (BioLegend) was added at a concentration of 10 μg/mL. For positive control, CD4^+^ T cells were stimulated with 50 ng/mL PMA (Merck) and 1 μg/mL ionomycin (Merck) for 2 h, and then brefeldin A (BioLegend) was added at a concentration of 10 μg/mL. CD4^+^ T cells were incubated for 14 h after brefeldin A addition before intracellular cytokine staining (Molodtsov et al., 2022). CD4^+^ T cells were washed with PBS and centrifuged. Cells were stained with anti-human CD3-FITC and CD4 antibodies for 15 min at 4°C. Then, cells were washed with PBS and fixed with 2% paraformaldehyde in PBS for 20 min at 4°C. Cells were washed and stained with an anti-human IFNγ antibody for 40 min at 4°C. Cells were analyzed using the NovoCyte flow cytometer (ACEA Biosciences).

Alternatively, CD4^+^ T cells were incubated with EVs at a 1:1 ratio in a 24-well plate (Corning) for 3 days in RPMI 1640 medium. After 3 days, the medium was changed to RPMI 1640 supplemented with 12 IU/mL recombinant IL-2 (STEMCELL Technologies). The cell culture medium supplemented with 12 IU/mL recombinant IL-2 was replenished every 2 days. The last medium change was absent for IL-2. At day 14, cell CD4^+^ T cells were counted and restimulated with EVs at a 1:1 ratio with culture medium changed to AIM-V medium supplemented with AlbuMAX in a 96-well round bottom plate. The intracellular IFNγ staining was performed under the same conditions as described.

### Differentiation of MoDCs from monocytes

Isolated PBMCs were re-suspended in RPMI 1640 supplemented with 10% FBS, 100 U/mL penicillin, 100 μg/mL streptomycin, 0.25 μg/mL amphotericin B, and 2 mM GlutaMAX (Gibco) and seeded in 25-cm^2^ cultural flasks (Corning) at a concentration of 6∗10^6^ cell/mL. After 2 h, the unbound cells were removed, and the media were changed to fresh, supplemented with growth factors—IL-4 (100 ng/mL) and GM-CSF (50 ng/mL) (STEMCELL Technologies), and then cultivated for 6 days with a change of half of the media volume every 2 days. After 6 days, the full medium volume was changed to a fresh portion with bacterial lipopolysaccharide (10 μg/mL) and cultivated for 24 h for DC maturation. MoDCs were analyzed for the expression of CD11c, CD80, and HLA-DR on the membrane surface using the NovoCyte flow cytometer (ACEA Biosciences).

### Statistical analysis

Statistical analysis was performed using GraphPad Prism 8.0 (GraphPad). The statistical test employed is denoted in each figure legend: * indicates *p* < 0.05, **indicates *p* < 0.01, *** indicates *p* < 0.001, and **** indicates *p* < 0.0001.

## Results

### Generation of EVs carrying CD80 and pMHC molecules

T-cell activation relies on two essential signals: one initiated by the interaction of TCR with pMHCs and the other facilitated by the CD28 stimulatory receptor, which binds the B7 costimulatory molecules (CD80/CD86) expressed on APCs. Notably, CD86 is a key ligand for CTLA-4, inducing an overall inhibitory effect, whereas CD80 is responsible for restraining CTLA-4 recycling (Kennedy et al., 2022). Therefore, we used transgenic cell lines engineered to express both CD80 molecules and pMHCs featuring variable peptides as a source of EVs. To ensure optimal presentation of the peptide on each MHC-II molecule of different alleles, we introduced a peptide of interest, tethered with an SG linker, to the beta chain of MHC-II—an approach distinct from exogenous peptide loading (Kozono et al., 1994; Li et al., 2022). The presence of clusters of specific pMHCs has been shown to possess superior potency in the activation of antigen-specific CD4^+^ T cells (Al-Aghbar et al., 2022).

**FIGURE 1:**
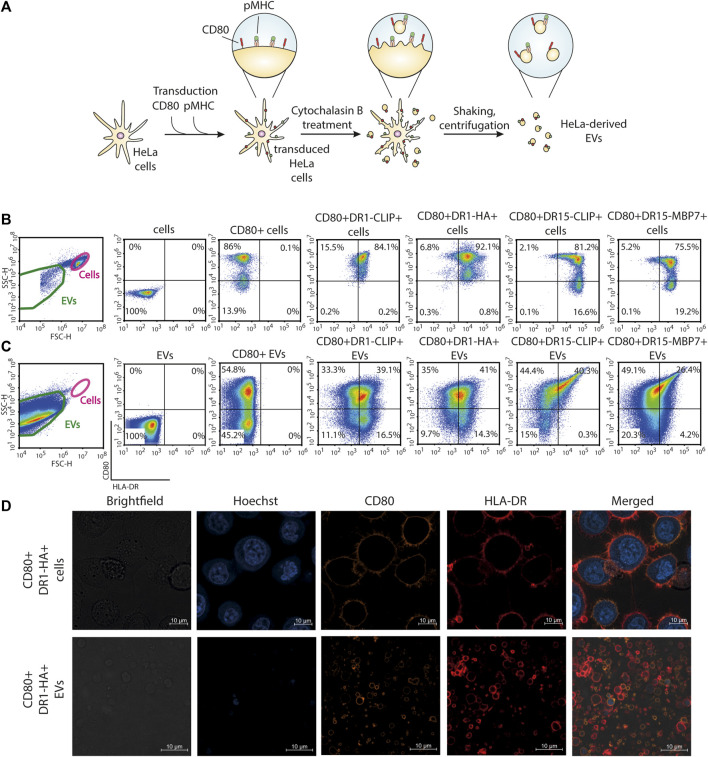
Generation of antigen-presenting CD80^+^HLA-DR^+^ EVs from the HeLa cell line. **(A)** Schematic representation of the main steps of the generation of EVs from HeLa cell lines carrying CD80 and pMHC molecules. **(B)** Surface staining for the expression of CD80 and HLA-DR molecules on HeLa cell lines. **(C)** Surface staining for the expression of CD80 and HLA-DR molecules on HeLa-derived EVs. Control non-transduced cells and transduced cells or derived EVs were stained with fluorescent antibodies. The analysis was carried out with flow cytometry. **(D)** Confocal imaging of CD80^+^DR1-HA^+^ cells and EVs. Nuclei were stained with Hoechst 33342 (blue), and CD80 and HLA-DR molecules were stained with PE-(orange) and APC-labeled (red) antibodies, respectively.

Although MoDCs are commonly employed for the presentation of antigens on MHC-II molecules due to their robust phagocytic activity (Schlitzer et al., 2015), the generation of MoDCs often yields a limited cell population. As an alternative, the immortalization of B cells, another frequently utilized approach for antigen-presentation studies, requires extended and intricate protocols for production (Topalian et al., 1994; Linnemann et al., 2015; Wang et al., 2023). In this study, we opted for HeLa cells for the generation of EVs, a widely used and versatile cell line enabling the fast generation of transgenic cell lines (Figure 1A). We created a panel of genetic constructs with two different MHC-II molecules: HLA-DR1 (HLA-DRB1*01:01) in complex with influenza A HA_306-318_ peptide and HLA-DR15 (HLA-DRB1*15:01) in complex with autoantigenic myelin basic protein MBP_85-99_ peptide. As a negative control, we developed a construct with a fragment of the invariant chain—CLIP peptide—for both the DR1 and DR15 complexes that does not elicit a T-cell response. A stepwise lentiviral transduction of HeLa cells with constructs encoding CD80 and specific pMHC molecules was performed (Figure 1B). All transduced HeLa cells exhibited more than 75% positivity for the expression of CD80 and pMHC.

Obtained transgenic HeLa CD80^+^DR^+^ cell lines were treated with cytochalasin B, which induces a swift disintegration of the actin cytoskeleton and the establishment of elongated tubular protrusions that could be effectively separated through agitation. The produced CD80^+^DR^+^ EVs were separated from cells with a series of benchtop centrifugations. This procedure resulted in the production of EVs that bear a replica of the membrane surface proteins expressed by the parent cell. Obtained EVs had a mean diameter of 2,400 nm (Ukrainskaya et al., 2021) and were readily detectable with flow cytometry. More than 25% of EVs demonstrated dual positivity for CD80 and pMHC (Figure 1C). Confocal imaging of HeLa cells and HeLa-derived EVs further verified the presence of HLA-DR and CD80 on the membrane surface (Figure 1D; Supplementary Figure S2).

### Modification of the Jurkat 76 TPR cell line

To evaluate the activation potential of the isolated EVs, we established a specialized cell line bearing cognate TCRs specific to tested pMHCs. To minimize potential experimental biases associated with the emergence of chimeric TCRs, we adopted the Jurkat 76 TPR (triple parameter reporter) cell line with green fluorescent protein (GFP) under transcription nuclear factor of activated T cell (NFAT) promoter, which lacks endogenous TCR alpha and beta chains (Rosskopf et al., 2018).

**FIGURE 2:**
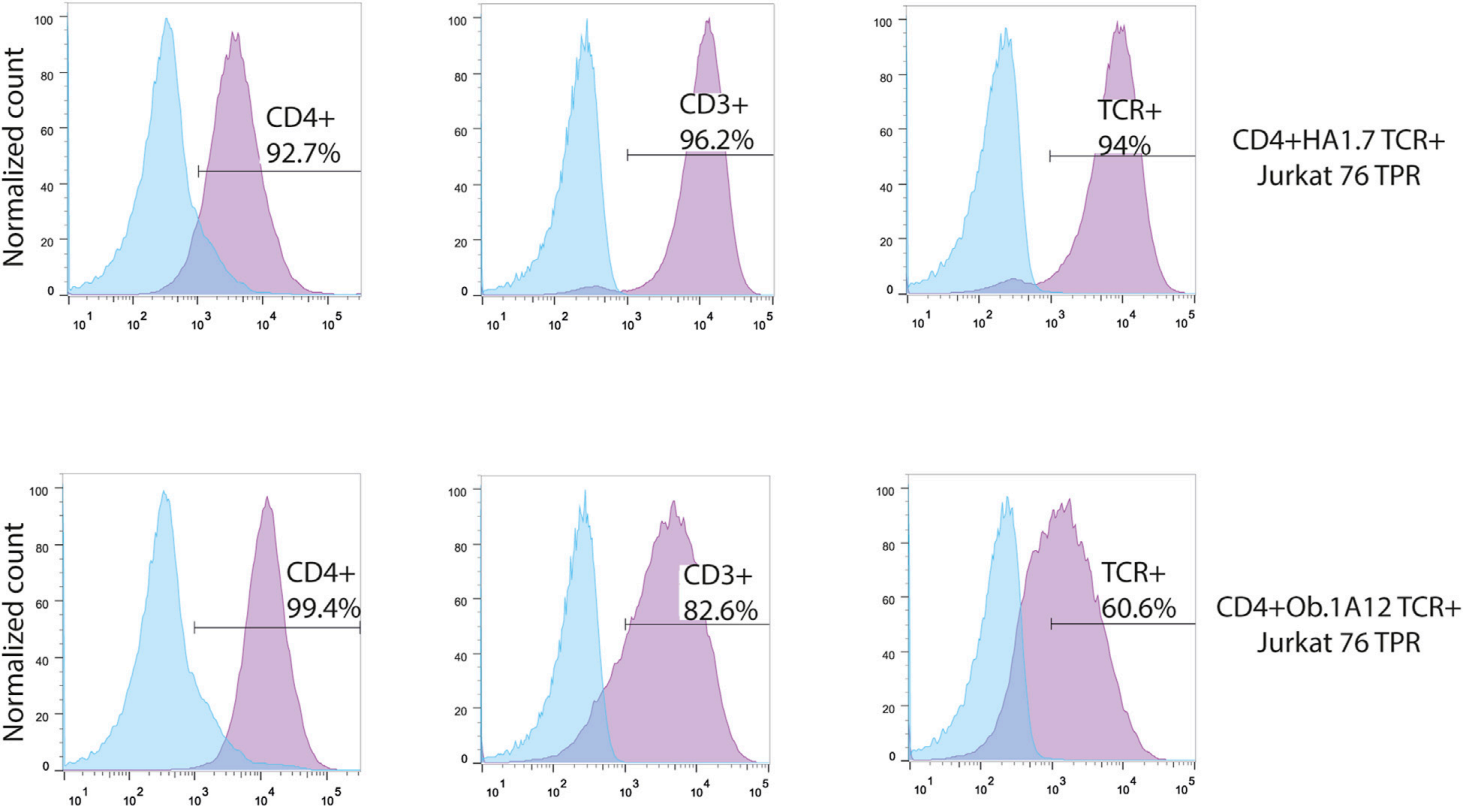
Transgenic Jurkat 76 TPR cell lines co-expressing CD4 and HA1.7 or Ob.1A12 TCRs. Surface staining for the expression of CD4, CD3, and TCR molecules on Jurkat 76 TPR cell lines transduced with lentiviruses coding for CD4 and TCR. The analysis was carried out with flow cytometry. Values indicate the percentage of positive antigen expression. Blue-shaded histograms represent non-transduced Jurkat 76 TPR cells stained with the designated fluorophore-conjugated antibody.

To augment the low endogenous expression of the CD4 molecule in Jurkat 76 TPR cells, we transduced DNA coding for this essential co-receptor (Figure 2). Subsequently, this transgenic cell line was further transduced by DNA coding for HA1.7 or Ob.1A12 TCRs, specific for HA_306-318_ peptide in the context of HLA-DR1 (CD4^+^HA1.7 TCR^+^ Jurkat 76 TPR) and MBP_85-99_ in the context of HLA-DR15 (CD4^+^Ob.1A12 TCR^+^ Jurkat 76 TPR), respectively, with cysteine substitutions in the constant domains to enhance chain pairing (Kuball et al., 2006). Notably, it was previously reported that HA1.7 exhibits a relatively high affinity for its cognate pMHC, whereas Ob.1A12 exhibits a lower affinity (K_D_>200 μM) attributable to an alternative positioning topology of the TCR (Sundberg et al., 2007).

### EVs harboring cognate CD80–pMHC activate transgenic CD4^+^TCR^+^ Jurkat 76 TPR cells in an antigen-specific manner

**FIGURE 3:**
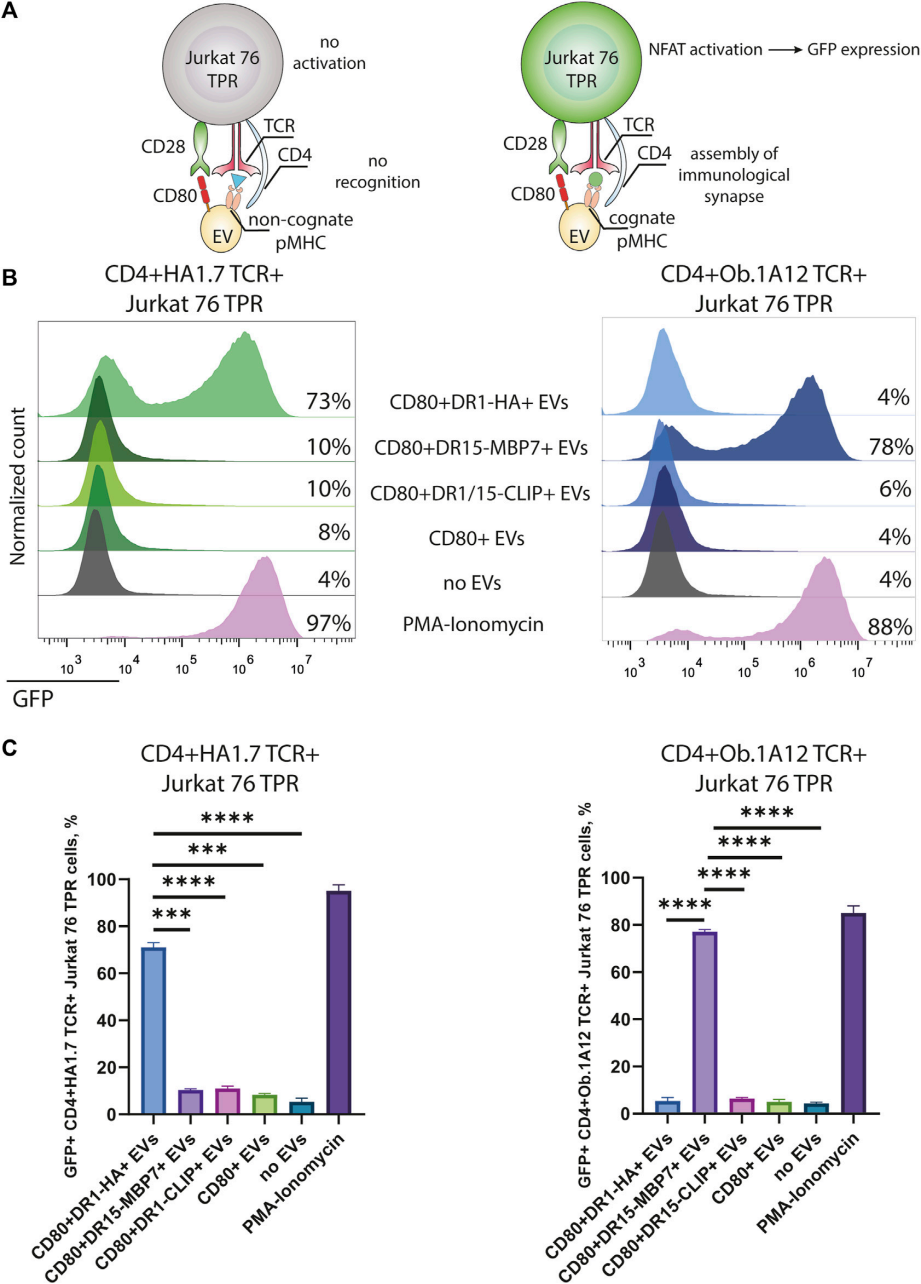
Stimulation of CD4^+^TCR^+^ Jurkat 76 TPR cells with antigen-presenting EVs. **(A)** Schematic representation of Jurkat 76 TPR activation due to the recognition of cognate pMHC with TCR. **(B)** CD4^+^HA1.7 TCR^+^ or CD4^+^Ob.1A12 TCR^+^ Jurkat 76 TPR cells were incubated with EVs for 16 h with a 5-fold excess of EVs. No stimulation (no EVs) was used as a negative control. Stimulation with PMA and ionomycin was used as the non-specific positive control. The analysis was carried out with flow cytometry. Values indicate the percentage of activated GFP-expressing Jurkat 76 TPR cells. Representative flow cytometry profiles are shown. **(C)** Percentage of GFP-positive CD4^+^TCR^+^ Jurkat 76 TPR cell lines exposed to various EVs are shown as the mean ± standard deviation (SD) of three experimental replicates. Statistical analysis was performed using Welch’s *t*-test.

Next, we assessed the specificity and amplitude of the activation potential of CD4^+^TCR^+^ Jurkat 76 TPR cell lines in response to CD80^+^DR^+^ EV exposure. The Jurkat 76 TPR cell line expresses GFP upon activation, facilitating our assessment of TCR specificity (Figure 3A). Our data revealed that Jurkat 76 TPR cell activation occurred exclusively when these cells were subjected to incubation with EVs bearing cognate pMHCs (Figures 3B, C). Specifically, CD4^+^HA1.7 TCR^+^ Jurkat 76 TPR cells exposed to a five-fold excess of CD80^+^DR1-HA^+^ EVs demonstrated a substantial 73% GFP-positive response. In contrast, incubation with EVs carrying either CD80 molecules (CD80^+^ EVs) alone or irrelevant pMHCs (CD80^+^DR1–CLIP^+^ or CD80^+^DR15–MBP7^+^ EVs) resulted in negligible activation, with less than 10% of GFP-positive cells. Similarly, the CD4^+^Ob.1A12 TCR^+^ Jurkat 76 TPR line exhibited activation exclusively in response to incubation with CD80^+^DR15–MBP7^+^ EVs. Moreover, the incubation with antigen-specific EVs resulted in the expression of CD69 activation markers for both CD4^+^HA1.7 TCR^+^ and CD4^+^Ob.1A12 TCR^+^ Jurkat 76 TPR (Supplementary Figure S3).

**FIGURE 4:**
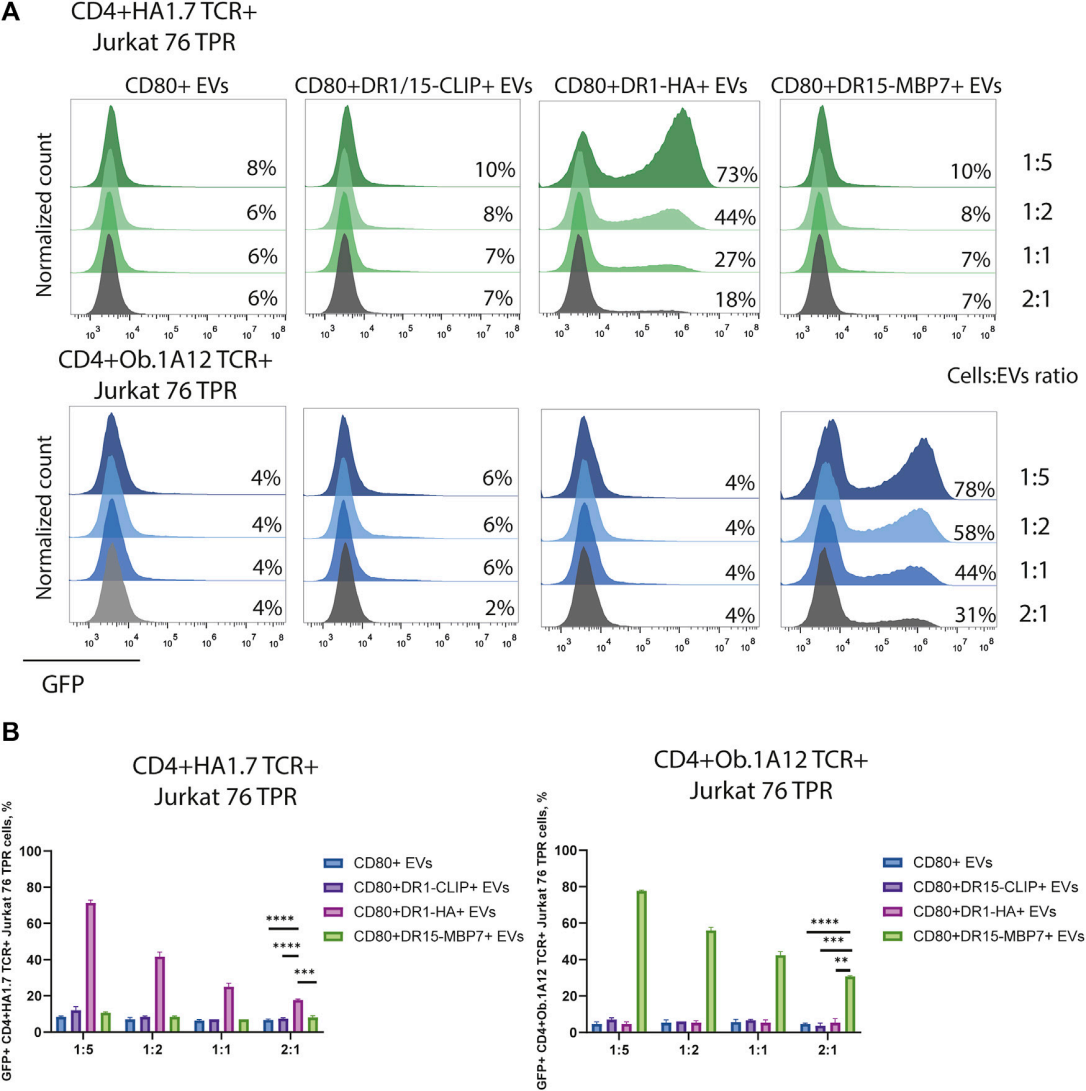
Stimulation of CD4^+^TCR^+^ Jurkat 76 TPR cells with EVs at different ratios. **(A)** CD4^+^HA1.7 TCR^+^ or CD4^+^Ob.1A12 TCR^+^ Jurkat 76 TPR cells were incubated with EVs for 16 h with cell:EV ratios of 1:5, 1:2, 1:1, and 2:1. The analysis was carried out with flow cytometry. Values indicate the percentage of activated GFP-expressing cells. Representative flow cytometry profiles are shown. **(B)** Percentage of GFP-positive CD4^+^TCR^+^ Jurkat 76 TPR cell lines exposed to various EVs are shown as the mean ± standard deviation of three experimental replicates. Statistical analysis was performed using Welch’s *t*-test.

The observed CD4^+^TCR^+^ Jurkat 76 TPR activation response exhibited dose-dependency (Figures 4A, B), as evidenced by levels of GFP-positive reporters in response to incubation with EVs in different ratios. The number of GFP-positive cells increased with the administration of a greater quantity of EVs. We noted that EVs carrying weakly interacting DR15–MBP7 complexes induced an even more potent activation response in CD4^+^Ob.1A12 TCR^+^ Jurkat 76 TPR cells compared to CD80^+^DR1-HA^+^ EVs interacting with CD4^+^HA1.7 TCR^+^ Jurkat 76 TPR in the same quantity. Additionally, we compared the activation capacity of CD80^+^DR1-HA^+^ EVs and MoDCs to activate CD4^+^HA1.7 TCR^+^ Jurkat 76 TPR cell lines (Supplementary Figure S4). EVs activated cell lines more potently than MoDCs, further confirming their activation ability.

Collectively, both high- and low-affinity TCRs demonstrated activation in response to EVs without inducing any discernible nonspecific background activation. We also demonstrated that pMHCs remained intact throughout the EV generation protocol involving cytochalasin B treatment and retained their capability to engage TCR. Consequently, our data demonstrate the high capacity of EVs to trigger T-cell responses.

### EVs induce the antigen-specific expansion of native CD4^+^ T cells from humans

Our data suggest that EVs boast a notable advantage as carriers of a substantial load of pMHCs and exhibit relative stability. These characteristics render them particularly suitable for the antigen-specific expansion of CD4^+^ T cells usually required for the study of T-cell responses originating from rare clones. Therefore, we aimed to investigate the feasibility of utilizing EVs to stimulate and expand human CD4^+^ T cells in an antigen-specific manner. To achieve this, we generated EVs bearing the Flu B HA_270-286_ epitope presented within the HLA-DR15 complex (CD80^+^DR15–FLU^+^ EVs). This specific epitope has been previously reported as an elicitor of anti-viral CD4^+^ T-cell responses in individuals bearing the HLA-DRB1*15:01 allele (Xiaomin et al., 2020).

**FIGURE 5:**
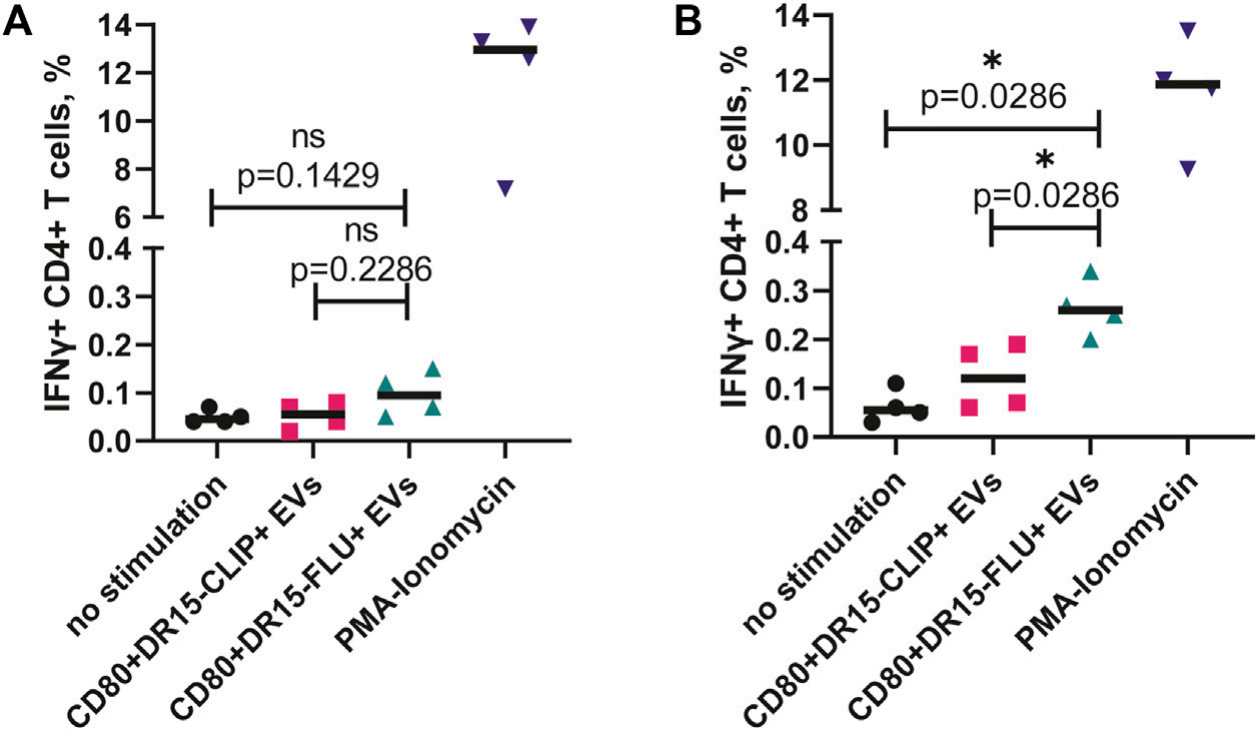
ntigen-specific expansion of human CD4^+^ T cells driven by antigen-presenting EVs. **(A)** Isolated CD4^+^ T cells from four HDs were incubated with EVs overnight before the assessment of IFNγ production. **(B)** Isolated CD4^+^ T cells from four HDs were incubated with CD80^+^DR15–FLU^+^ EVs for 3 days and then expanded for a total of 14 days with further restimulation with CD80^+^DR15–CLIP^+^ or CD80^+^DR15–FLU^+^ EVs. No stimulation was used as a negative control. Stimulation with PMA and ionomycin was used as the positive control. Each dot represents the mean value of three experimental replicates. Statistical analysis was performed using the Mann–Whitney test.

We isolated CD4^+^ T cells from four healthy individuals with a confirmed heterozygous HLA-DRB1*15:01-positive haplotype. First, isolated cells were incubated with EVs for 16 h and then fixed and intracellularly stained for the production of IFNγ. The difference in the percentage of IFNγ^+^ cells between CD80^+^DR15–FLU^+^ and CD80^+^DR15–CLIP^+^ EVs was not significant (Figure 5A). Alternatively, isolated cells were subjected to stimulation with CD80^+^DR15–FLU^+^ EVs for 3 days, followed by a subsequent 10-day expansion with a periodical addition of IL-2 to further maintain T-cell growth. On the 10th day, T cells were subjected to secondary stimulation. They were exposed to CD80^+^DR15–FLU^+^ or CD80^+^DR15–CLIP^+^ EVs for an additional 16 h. The difference in the percentage of IFNγ^+^ cells between CD80^+^DR15–FLU^+^ or CD80^+^DR15–CLIP^+^ EVs was statistically significant (*p* = 0.0286) (Figure 5B; Supplementary Figure S5). Collectively, our findings illustrate the efficacy of EVs in selectively promoting the proliferation of antigen-specific CD4^+^ T cells, enabling the detection of antigen-specific responses from rare T-cell clones.

## Discussion

In this study, we developed antigen-presenting EVs carrying pMHCs of interest and CD80 costimulatory molecules, which were employed for the stimulation and expansion of CD4^+^ T cells. The obtained EVs exhibited the capacity to activate both immortalized T-cell lines and primary human CD4^+^ T cells.

EVs are naturally secreted by all cell types and are commonly derived from the conditioned media of cultured cells. Various techniques, including physical and chemical stimulation or genetic manipulations, have been employed to enhance EV properties (Syromiatnikova et al., 2022). Standard isolation protocols often involve ultracentrifugation, precipitation, or chromatography for EV extraction (Ng et al., 2022). Alternatively, techniques such as sonication or nitrogen cavitation have been utilized to generate nanoparticles that mimic EVs (artificial EVs) and replicate the cell surface characteristics of naturally derived microvesicles (Gao et al., 2016; Thamphiwatana et al., 2017). In our study, we produced artificial EVs using cytochalasin B, which is a widespread chemical agent enabling the straightforward generation of a large quantity of EVs (Pick et al., 2005; Gomzikova et al., 2020). These HeLa-derived EVs carry membrane-associated proteins, similar to natural microvesicles, but are slightly larger in size (Théry et al., 2009). The size of HeLa-derived vesicles facilitates their quantification with flow cytometry and the assessment of surface markers. Furthermore, HeLa-obtained EVs have been reported to be stable and can be stored for several months at −80°C, preserving surface markers (Ukrainskaya et al., 2021).

Previous studies showed that natural EVs from APCs can present antigens to T cells (Raposo et al., 1996). However, exosomes with a diameter of 60–100 nm from the conditioned media carrying HLA-DR1–HA_306-318_ complexes weakly activated antigen-specific T cells, in contrast to cross-linking on latex beads or internalization with DCs (Vincent-Schneider et al., 2002). This may be attributed to the low clusterization level of pMHCs, which is insufficient to activate T cells due to the small size of EVs. To replicate the surface characteristics of natural EVs derived from APCs, we engineered EVs to carry both the CD80 costimulatory molecule and pMHCs, similar to professional APCs. Previously, genetic modification of artificial APCs with CD80 enabled expansion of antigen-specific CD4^+^ T cells (Butler et al., 2010; Garnier et al., 2016). In studies on antigen presentation, antigenic peptides are typically either loaded exogenously or genetically implemented, often in conjunction with trafficking motifs such as the invariant chain or MHC class-I trafficking domain (MITD) protein, to enhance antigen presence in endosome compartments (Lee and Meyerson, 2021; Wang et al., 2023). However, it has been reported that the genetic implementation of antigenic minigenes can elicit lower reactivity compared to the exogenous loading of antigenic fragments (Parkhurst et al., 2019). Exogenous loading of peptides may also require competitive displacement of the peptide already presented on MHC-II, which can be problematic when exogenous loading is required for low-affinity peptides that may still be presented and elicit T-cell responses under physiological conditions (Stadinski et al., 2010; Yang et al., 2014; Ishina et al., 2023). In our system, each peptide of interest is covalently bound to the beta chain of the MHC-II molecule, allowing for the presentation of low-affinity peptides. Previously, the presentation of peptides covalently linked to the beta chain of MHC-II was successfully achieved on the cell surface (Yang et al., 2014; Obarorakpor et al., 2023). Consequently, the relatively large diameter of HeLa-derived EVs and the high surface concentration of CD80 and pMHCs of interest make EVs capable of individual activation of antigen-specific CD4^+^ T cells without carrier beads or cells. Despite the much lower affinity of Ob.1A12 TCR to cognate pMHC, the activation of CD4^+^Ob.1A12 TCR^+^ Jurkat 76 TPR was higher compared to CD4^+^HA1.7 TCR^+^ Jurkat 76 TPR stimulated with EVs carrying the HA epitope. This further confirms the sufficient density of antigenic epitopes on HeLa-derived EVs, which enables the engagement of even low-affinity TCRs in promoting activation of T cells.

Due to the lack of proliferation, EVs offer promise for applications in antigen-specific T-cell expansion. Several approaches exist for expanding T cells, including nonspecific stimulation of CD3/CD28 or PHA, to polyclonally expand T cells with the aim of expanding antigen-specific T cells as well (Trickett and Kwan, 2003; Geiger et al., 2009). However, anti-CD3 and anti-CD28 antibodies may lead to suboptimal expansion due to activation-induced cell death and a decrease in viability (Zappasodi et al., 2008; Fadel et al., 2014). Alternatively, T cells can be enriched for activation markers (CD154 or CD137) by incubating them with antigens and then further expanded (Lee et al., 2020; Abdirama et al., 2021). Typically, to specifically expand antigen-specific T cells, APCs presenting in PBMCs or MoDCs are loaded with antigens and incubated for several days with the addition of growth factors, including IL-2, IL-7, or IL-15 (Cimen Bozkus et al., 2021). However, MoDCs may be dysfunctional, for example, due to the presence of the tumor microenvironment (Veglia and Gabrilovich, 2017). Moreover, several antigen-specific expansion approaches using artificial APCs (aAPCs) and cell-free systems have been described (Ichikawa et al., 2020; Isser et al., 2022; Sun et al., 2022). The application of APCs or aAPCs introduces difficulties related to proliferation and the consumption of growth factors, while cell-free approaches require the laborious conjugation of pMHCs or costimulatory molecules. Ensuring the absence of an exhausted phenotype in expanded T cells is imperative for the subsequent functional characterization of the expanded population. Notably, HeLa-derived EVs, generated using cytochalasin B, as previously reported, demonstrated the lowest expression of TIGIT on CAR-T cells after a 2-week period, in contrast to the effects observed with Dynabeads and IL-2 during expansion (Ukraiskaya et al., 2021). However, it should be noted that prolonged and continuous antigen stimulation may lead to the development of an exhausted T cell phenotype (Han et al., 2010; Ukrainskaya et al., 2023). Accordingly, we stimulated CD4^+^ T cells with EVs once during the antigen-specific expansion and successfully expanded CD4^+^ T cells specific to the Flu B HA_270-286_ viral epitope.

Our technique describing the elaboration of antigen-presenting EVs capable of activating and expanding CD4^+^ T cells (Supplementary Figure S6) provides extensions of several immunotherapeutic protocols: i) the developed EVs can be employed for the expansion and investigation of CD4^+^ T cell phenotypes, ii) transcriptomic analysis, and iii) cytokine production assessment. Additionally, iv) the analysis of expanded T cells can be used to identify TCR sequences, particularly relevant to cancer and autoimmune diseases, where TCRs often bind to respective pMHCs with low affinity, making their identification challenging using conventional tetramer techniques.

## Data Availability

The original contributions presented in the study are included in the article/Supplementary Material; further inquiries can be directed to the corresponding authors.
