# Supplementary material for: Genetically engineered CD80–pMHC-harboring extracellular vesicles for antigen-specific CD4+ T-cell engagement
Source: Front Bioeng Biotechnol. 2024 Jan 17;11:1341685. doi: 10.3389/fbioe.2023.1341685 (PMC10833362; doi:10.3389/fbioe.2023.1341685)
Supplement: Supplementary file 1 [file DataSheet1.docx]

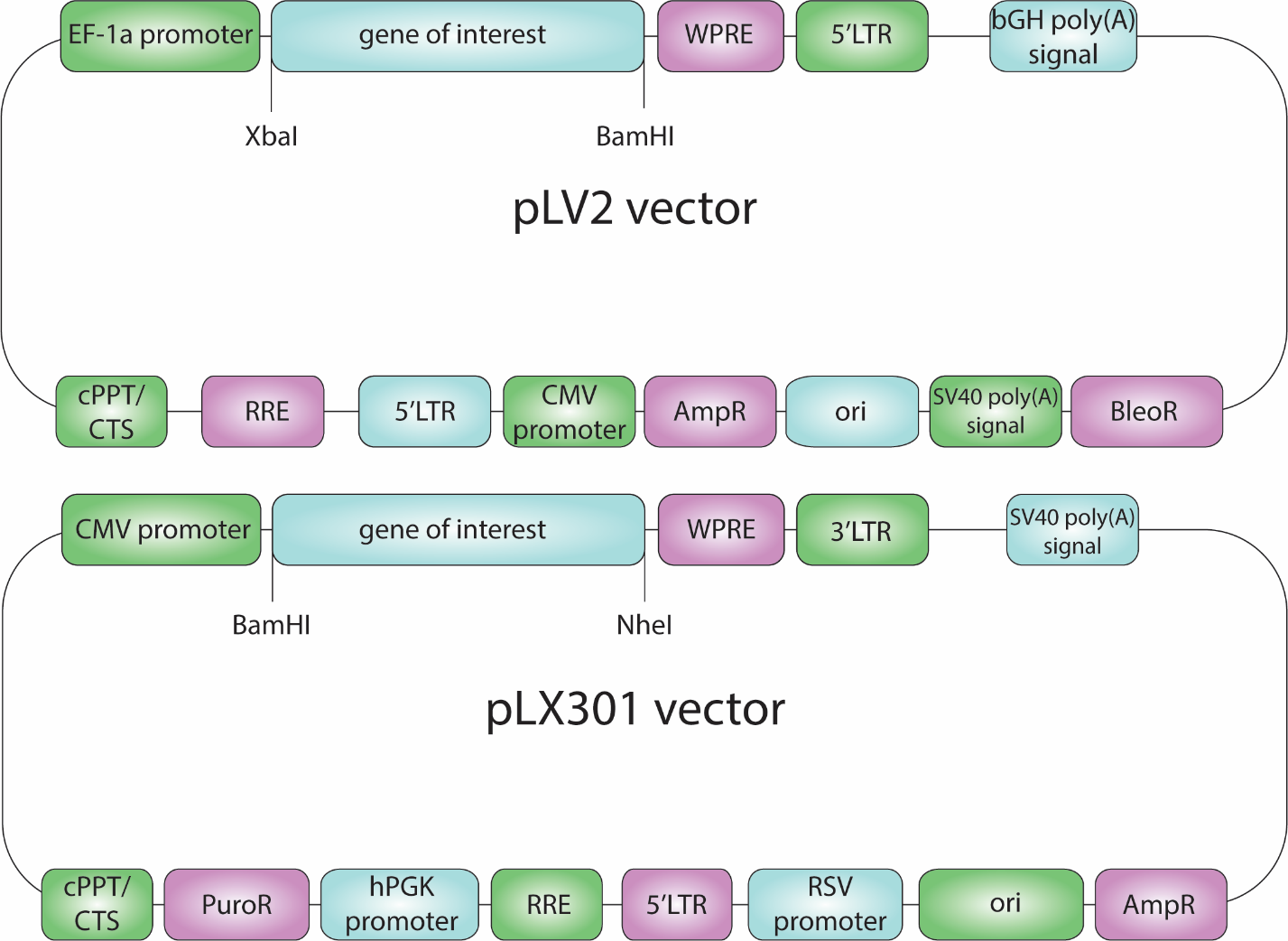


**Supplementary figure 1. Vector maps of pLV2 and pLX301 genetic constructs.** Gene of interest is expressed under the control of EF-1a or CMV promoter in pLV2 or pLX301 lentiviral vectors, respectively.


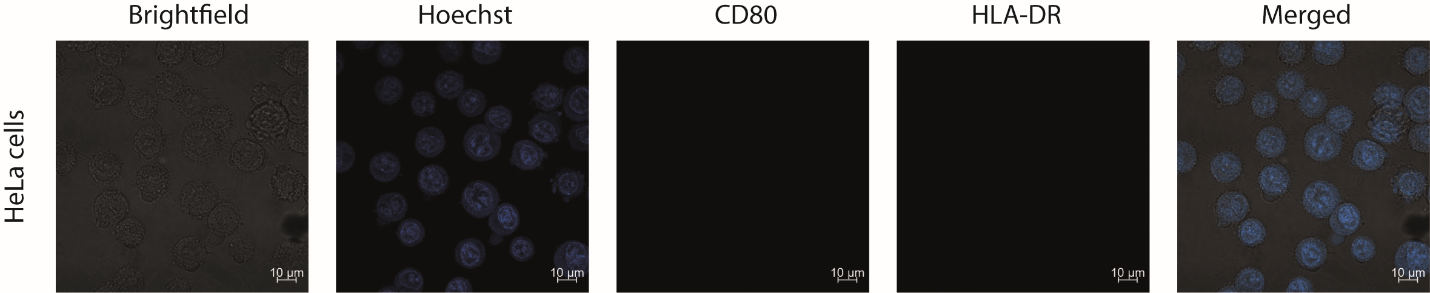


**Supplementary figure 2. Confocal imaging of non-transduced with CD80 and HLA-DR HeLa cells.** Nuclei were stained with Hoechst 33342 (blue), CD80 and HLA-DR molecules were stained with PE- (orange) and APC-labeled (red) antibodies, respectively.


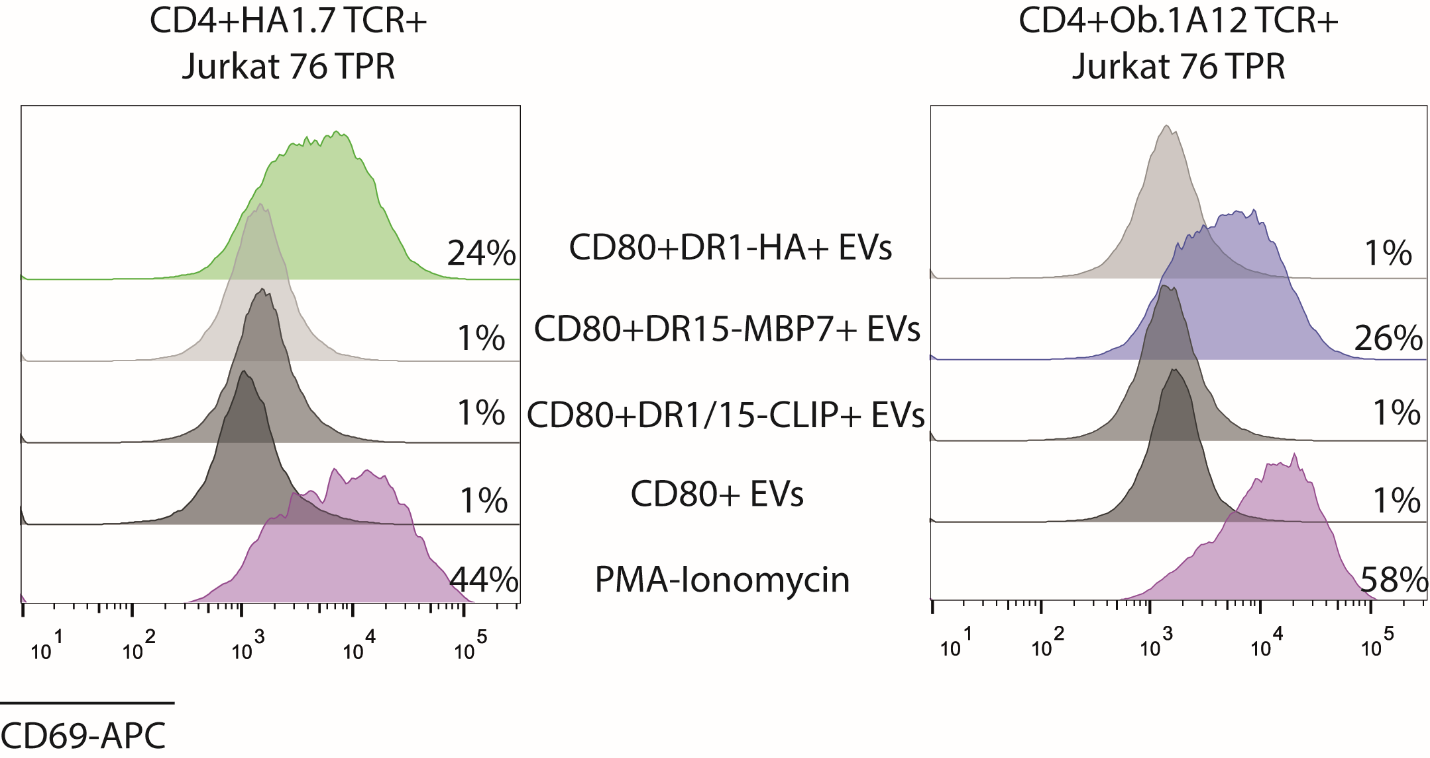


**Supplementary figure 3. Assessment of CD69 expression on activated CD4^+^TCR^+^ Jurkat 76 TPR cells.** CD4^+^HA1.7 TCR^+^ or CD4^+^Ob.1A12 TCR^+^ Jurkat 76 TPR cells were incubated with 5-fold excess of EVs for 16 hours. Stimulation with PMA and Ionomycin was used as non-specific positive control. The analysis was carried out with flow cytometry. Values indicate the percentage of activated CD69-expressing CD4^+^TCR^+^ Jurkat 76 TPR cells.


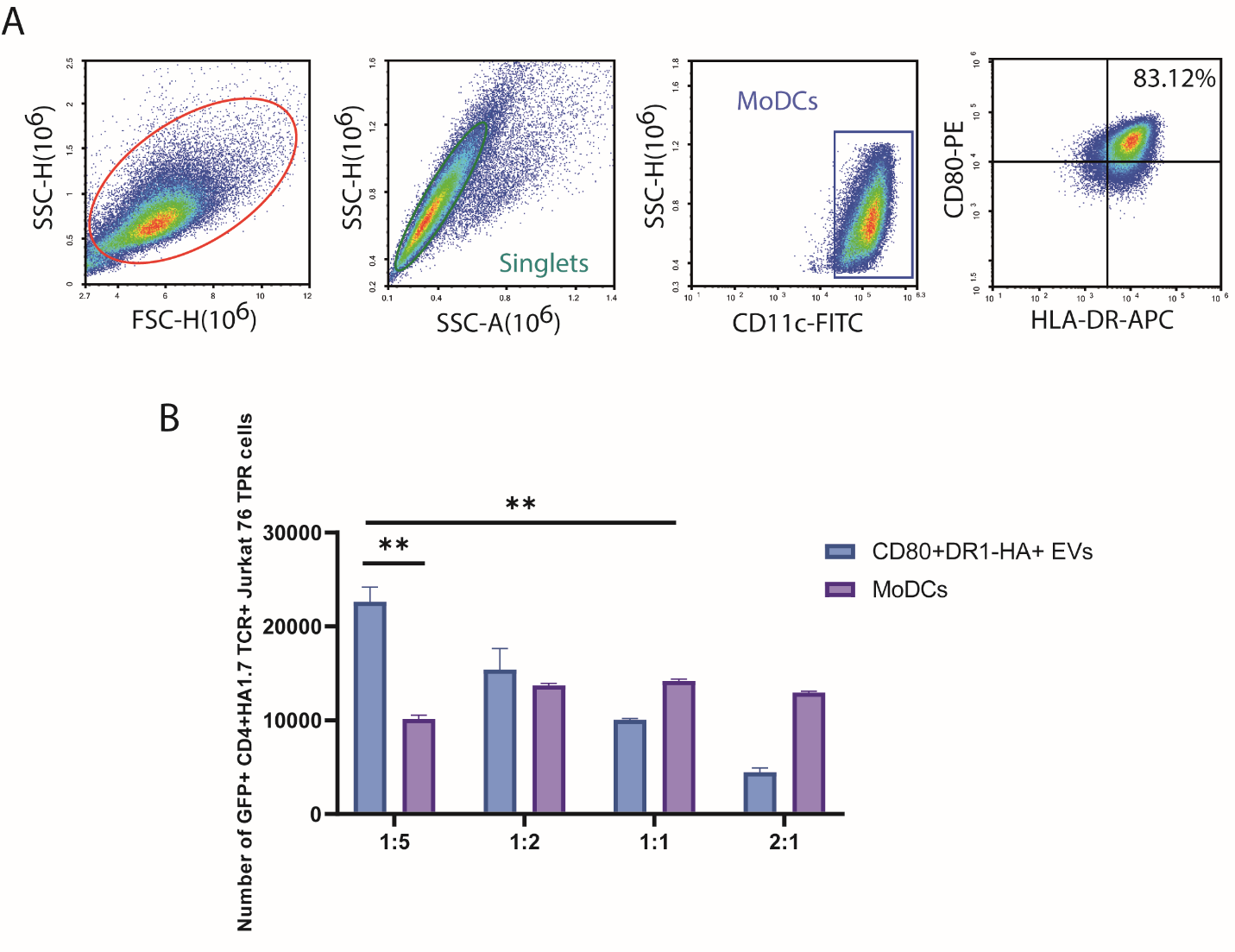


**Supplementary figure 4. Activation of CD4^+^HA1.7 TCR^+^ Jurkat 76 TPR cell line with EVs and MoDCs.** (A) Surface staining for the expression of CD11c, CD80, and HLA-DR of MoDCs of HLA-DRB1*01:01-positive individual. The analysis was carried out with flow cytometry. (B) CD4^+^HA1.7 TCR^+^ Jurkat 76 TPR cells were incubated with CD80^+^DR1-HA^+^ EVs or MoDCs for 16 hours with Jurkat 76 TPR cells:EVs or MoDCs ratio of 1:5, 1:2, 1:1, and 2:1. The analysis was carried out with flow cytometry. Number of GFP-positive CD4^+^HA1.7 TCR^+^ Jurkat 76 TPR cells are shown as mean ± standard deviation (SD) of three experimental replicates. Statistical analysis was performed using Welch’s t test


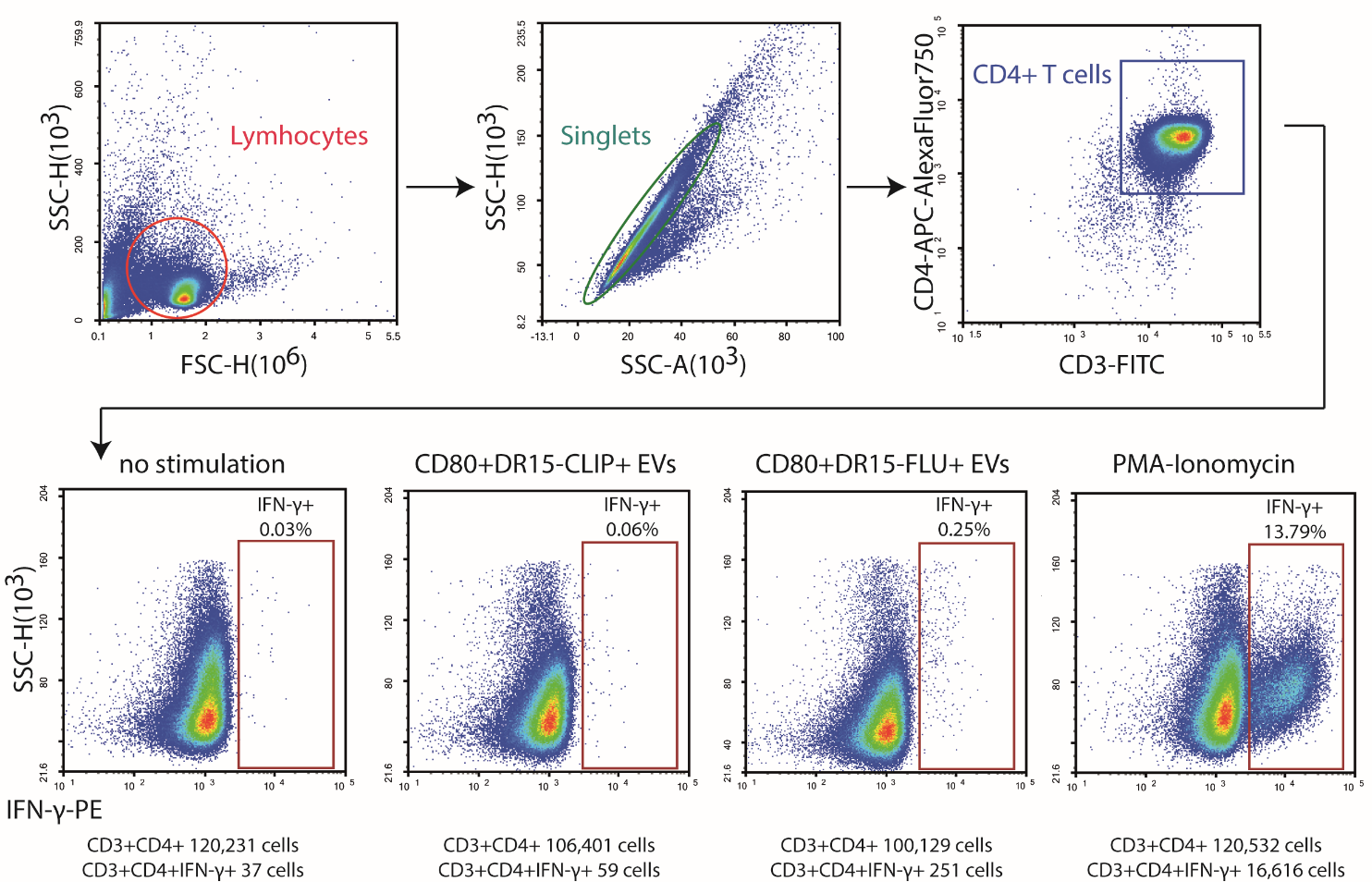


**Supplementary figure 5. Representative flow cytometry plots assessing production of IFN-γ by CD4^+^ T cells expanded with CD80^+^DR15-FLU^+^ EVs.** Isolated CD4^+^ T cells from HLA-DRB1*15:01-positive donors were expanded with CD80^+^DR15-FLU^+^ EVs with the addition of IL-2 and then restimulated with CD80^+^DR15-CLIP^+^ EVs or CD80^+^DR15-FLU^+^ EVs or PMA-Ionomycin. Expression of IFN-γ was examined with flow cytometry.


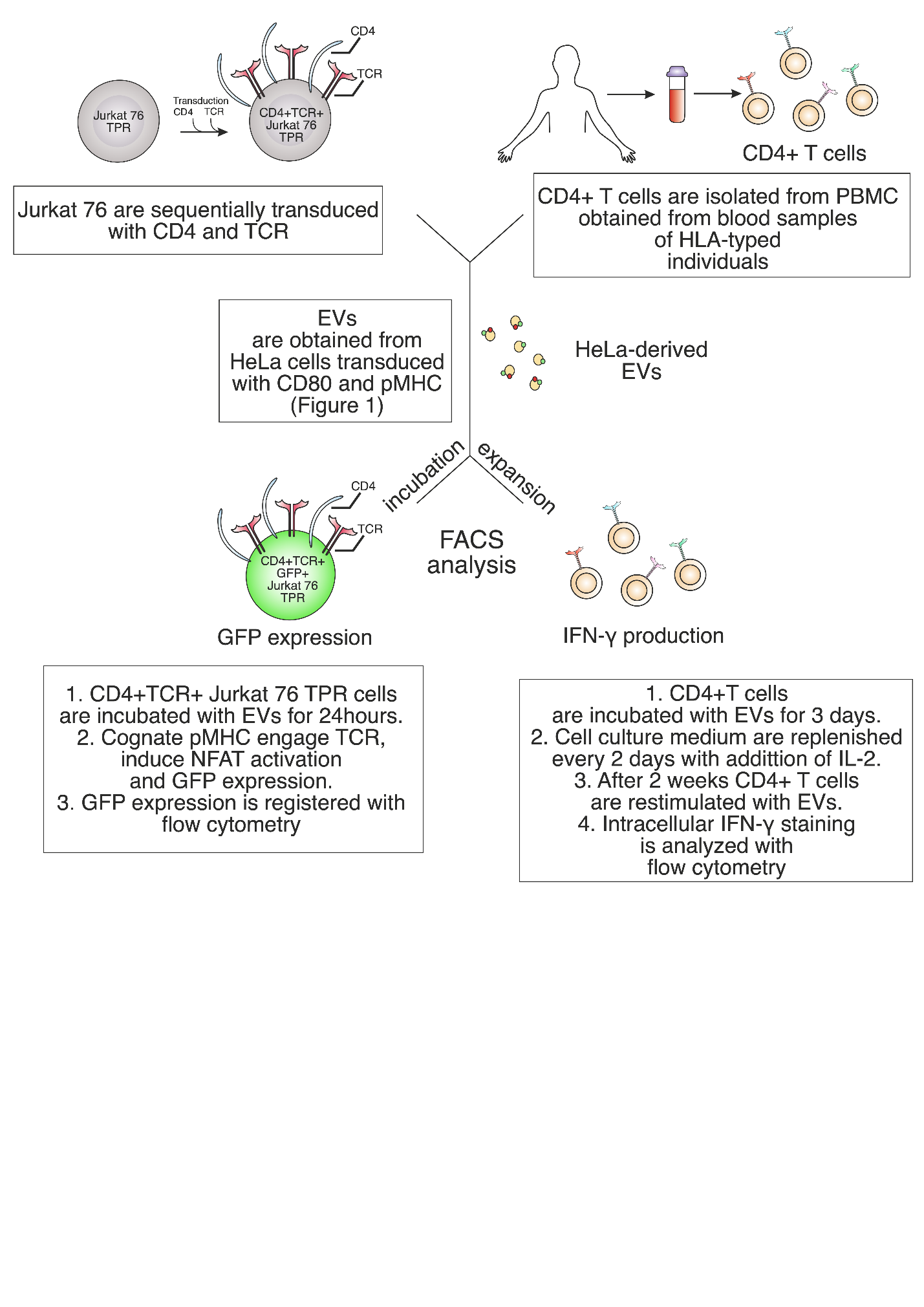


**Supplementary figure 6. Schematic diagram illustrating application of EVs for activation and expansion of CD4^+^ T cells.** Jurkat 76 TPR cell line is sequentially transduced with CD4 and TCR. Human CD4^+^ T cells are isolated from PBMC of HLA-typed donors. EVs are obtained from HeLa cell line modified to express CD80 and pMHC complex of interest (Figure 1). The activation of Jurkat 76 TPR cell line is assessed by GFP expression registered with flow cytometry. Human CD4^+^ T cells are expanded with EVs and IFN-γ production is evaluated with flow cytometry.

**Supplementary table 1. Amino acid sequences of proteins corresponding to genes used in the study.**

| **Name** | **Sequence (aa)** |
| --- | --- |
| CD4 | MNRGVPFRHLLLVLQLALLPAATQGKKVVLGKKGDTVELTCTASQKKSIQFHWKNSNQIKILGNQGSFLTKGPSKLNDRADSRRSLWDQGNFPLIIKNLKIEDSDTYICEVEDQKEEVQLLVFGLTANSDTHLLQGQSLTLTLESPPGSSPSVQCRSPRGKNIQGGKTLSVSQLELQDSGTWTCTVLQNQKKVEFKIDIVVLAFQKASSIVYKKEGEQVEFSFPLAFTVEKLTGSGELWWQAERASSSKSWITFDLKNKEVSVKRVTQDPKLQMGKKLPLHLTLPQALPQYAGSGNLTLALEAKTGKLHQEVNLVVMRATQLQKNLTCEVWGPTSPKLMLSLKLENKEAKVSKREKAVWVLNPEAGMWQCLLSDSGQVLLESNIKVLPTWSTPVQPMALIVLGGVAGLLLFIGLGIFFCVRCRHRRRQAERMSQIKRLLSEKKTCQCPHRFQKTCSPI |
| CD80 | MGHTRRQGTSPSKCPYLNFFQLLVLAGLSHFCSGVIHVTKEVKEVATLSCGHNVSVEELAQTRIYWQKEKKMVLTMMSGDMNIWPEYKNRTIFDITNNLSIVILALRPSDEGTYECVVLKYEKDAFKREHLAEVTLSVKADFPTPSISDFEIPTSNIRRIICSTSGGFPEPHLSWLENGEELNAINTTVSQDPETELYAVSSKLDFNMTTNHSFMCLIKYGHLRVNQTFNWNTTKQEHFPDNLLPSWAITLISVNGIFVICCLTYCFAPRCRERRRNERLRRESVRPV |
| HA1.7 TCR | MGIRLLCRVAFCFLAVGLVDVKVTQSSRYLVKRTGEKVFLECVQDMDHENMFWYRQDPGLGLRLIYFSYDVKMKEKGDIPEGYSVSREKKERFSLILESASTNQTSMYLCASSSTGLPYGYTFGSGTRLTVVEDLKNVFPPEVAVFEPSEAEISHTQKATLVCLATGFFPDHVELSWWVNGKEVHSGVCTDPQPLKEQPALNDSRYCLSSRLRVSATFWQNPRNHFRCQVQFYGLSENDEWTQDRAKPVTQIVSAEAWGRADCGFTSVSYQQGVLSATILYEILLGKATLYAVLVSALVLMAMVKRKDFEGRGSLLTCGDVEENPGPMLLLLVPVLEVIFTLGGTRAQSVTQLGSHVSVSEGALVLLRCNYSSSVPPYLFWYVQYPNQGLQLLLKYTSAATLVKGINGFEAEFKKSETSFHLTKPSAHMSDAAEYFCAVSESPFGNEKLTFGTGTRLTIIPYIQNPDPAVYQLRDSKSSDKSVCLFTDFDSQTNVSQSKDSDVYITDKCVLDMRSMDFKSNSAVAWSNKSDFACANAFNNSIIPEDTFFPSPESSCDVKLVEKSFETDTNLNFQNLSVIGFRILLLKVAGFNLLMTLRLWSS |
| Ob.1A12 TCR | MGLLLLLLGPGSGLGAVVSQHPSRVICKSGTSVKIECRSLDFQATTMFWYRQFPKQSLMLMATSNEGSKATYEQGVEKDKFLINHASLTLSTLTVTSAHPEDSSFYICSARDLTSGANNEQFFGPGTRLTVLEDLKNVFPPEVAVFEPSEAEISHTQKATLVCLATGFFPDHVELSWWVNGKEVHSGVCTDPQPLKEQPALNDSRYCLSSRLRVSATFWQNPRNHFRCQVQFYGLSENDEWTQDRAKPVTQIVSAEAWGRADCGFTSVSYQQGVLSATILYEILLGKATLYAVLVSALVLMAMVKRKDFEGRGSLLTCGDVEENPGPMETLLGVSLVILWLQLARVNSQQGEEDPQALSIQEGENATMNCSYKTSINNLQWYRQNSGRGLVHLILIRSNEREKHSGRLRVTLDTSKKSSSLLITASRAADTASYFCATDTTSGTYKYIFGTGTRLKVLAYIQNPDPAVYQLRDSKSSDKSVCLFTDFDSQTNVSQSKDSDVYITDKCVLDMRSMDFKSNSAVAWSNKSDFACANAFNNSIIPEDTFFPSPESSCDVKLVEKSFETDTNLNFQNLSVIGFRILLLKVAGFNLLMTLRLWSS |
| pMHC (DR1) | MVCLKLPGGSCMTALTVTLMVLSSPLALA-peptide-GGGGSGGGGSGGGGSGDTRPRFLWQLKFECHFFNGTERVRLLERCIYNQEESVRFDSDVGEYRAVTELGRPDAEYWNSQKDLLEQRRAAVDTYCRHNYGVGESFTVQRRVEPKVTVYPSKTQPLQHHNLLVCSVSGFYPGSIEVRWFRNGQEEKAGVVSTGLIQNGDWTFQTLVMLETVPRSGEVYTCQVEHPSVTSPLTVEWRARSESAQSKMLSGVGGFVLGLLFLGAGLFIYFRNQKGHSGLQPTGFLSVYKGEGRGSLLTCGDVEENPGPSGMAISGVPVLGFFIIAVLMSAQESWAIKEEHVIIQAEFYLNPDQSGEFMFDFDGDEIFHVDMAKKETVWRLEEFGRFASFEAQGALANIAVDKANLEIMTKRSNYTPITNVPPEVTVLTNSPVELREPNVLICFIDKFTPPVVNVTWLRNGKPVTTGVSETVFLPREDHLFRKFHYLPFLPSTEDVYDCRVEHWGLDEPLLKHWEFDAPSPLPETTENVVCALGLTVGLVGIIIGTIFIIKGLRKSNAAERRGPL |
| pMHC (DR15) | MVCLKLPGGSCMTALTVTLMVLSSPLALA-peptide-GGGGSGGGGSGGGGSGDTRPRFLWQPKRECHFFNGTERVRFLDRYFYNQEESVRFDSDVGEFRAVTELGRPDAEYWNSQKDILEQARAAVDTYCRHNYGVVESFTVQRRVQPKVTVYPSKTQPLQHHNLLVCSVSGFYPGSIEVRWFLNGQEEKAGMVSTGLIQNGDWTFQTLVMLETVPRSGEVYTCQVEHPSVTSPLTVEWRARSESAQSKMLSGVGGFVLGLLFLGAGLFIYFRNQKGHSGLQPTGFLSVYKGEGRGSLLTCGDVEENPGPSGMAISGVPVLGFFIIAVLMSAQESWAIKEEHVIIQAEFYLNPDQSGEFMFDFDGDEIFHVDMAKKETVWRLEEFGRFASFEAQGALANIAVDKANLEIMTKRSNYTPITNVPPEVTVLTNSPVELREPNVLICFIDKFTPPVVNVTWLRNGKPVTTGVSETVFLPREDHLFRKFHYLPFLPSTEDVYDCRVEHWGLDEPLLKHWEFDAPSPLPETTENVVCALGLTVGLVGIIIGTIFIIKGLRKSNAAERRGPL |
| Influenza A HA_306-318_ peptide | PKYVKQNTLKLAT |
| MBP_85-99_ peptide | ENPVVHFFKNIVTPR |
| Flu B HA_270-286_ peptide | GKTGTIVYQRGVLLPQK |
| CLIP peptide | PVSKMRMATPLLMQA |
